# Optimizing Yeast Homologous Recombination for Splicing Large Coronavirus Genome Fragments

**DOI:** 10.3390/ijms252413742

**Published:** 2024-12-23

**Authors:** Guoqing Xiong, Xuan Huang, Ao Hu, Zhixin Meng, Jiazhen Cui, Yuzhong Feng, Zhili Chen, Yuanyuan Lu, Qi Yang, Gang Liu

**Affiliations:** 1 Institutes of Physical Science and Information Technology, Anhui University, Hefei 230000, China; 2 Academy of Military Medical Sciences, Beijing 100850, China; 3 School of Life Science, Hebei University, Baoding 071000, China; 4 College of Life Sciences, Inner Mongolia Agricultural University, Hohhot 010011, China

**Keywords:** synthetic biology, reverse genetics, yeast homologous recombination, DNA assembly, automated splicing

## Abstract

Reverse genetics is a useful tool for studying viruses and developing vaccines for coronaviruses. However, constructing and manipulating the coronavirus genome in *Escherichia coli* can be time-consuming and challenging due to its large size and instability. Homologous recombination, a genetic manipulation mechanism found in organisms, is essential for DNA repair, gene recombination, and genetic engineering. In yeast, particularly Saccharomyces cerevisiae, homologous recombination technology is commonly used for constructing gene expression plasmids and genome editing. In this study, we successfully split and spliced a 30 kb viral genome fragment using yeast homologous recombination. By optimizing the program parameters, such as homologous arm lengths and fragment-to-vector ratios, we achieved a splicing efficiency of up to 97.9%. The optimal parameters selected were a 60 bp homologous sequence size and a vector fragment ratio of 1:2:2:2:2:2 for yeast homologous recombination of large DNA fragments.

## 1. Introduction

In recent years, the world has experienced several viral pandemics, such as Zika virus [1,2], novel coronavirus (SARS-CoV-2) [3,4], and mpox [5], which have had a significant impact on human health and social development.

Reverse genetics synthesis is a crucial tool in synthetic biology, allowing for the rapid synthesis or modification of viruses without being restricted by their source. This method has greatly advanced virus detection and treatment by enabling the addition of tags or fluorescence [6]. The synthesis and assembly of DNA fragments are key components of reverse genetics. Currently, the de novo synthesis of DNA fragments is limited in length, requiring enzymatic assembly of oligonucleotide fragments or in vivo assembly for longer genes or genomes.

Two commonly used oligonucleotide assembly methods are ligase chain reaction (LCR) and polymerase cycling assembly (PCA) [7]. Previous methods for splicing double-stranded DNA after oligonucleotide assembly relied on sticky ends generated by restriction endonucleases [8]. However, newer assembly methods using nucleases, DNA polymerases, and ligases have eliminated the need for restriction endonucleases. These methods involve assembly by generating homologous single-chain complementary ends. Efficient and simple assembly methods include SLIC (sequence- and ligation-independent cloning) [9], LCR (ligase chain reaction) [10], SLiCE (seamless ligation cloning extract) [11], CPEC (circular polymerase extension cloning) [12], and Gibson assembly [13].

As the size of DNA fragments increases, it becomes more difficult to maintain stability in vitro, and the assembly of large fragments relies heavily on the recombination system within the organism. *Escherichia coli*, *Bacillus subtilis*, and yeast are commonly used host cells for assembling long DNA fragments in vivo [14]. By recombining with bacterial artificial chromosomes (BACs) [15], the *Bacillus subtilis* genome (BGM) [16], or yeast artificial chromosomes (YACs) [17], these host cells can effectively carry large DNA fragments, with the BGM capable of cloning fragments exceeding 3 Mb [18]. Yeast in particular has a higher rate of homologous recombination and good compatibility with long fragments, making it the preferred cell for assembling multiple DNA fragments simultaneously. Various application methods have been developed based on this system [19,20,21]. For example, Gibson et al. successfully assembled 25 DNA fragments in brewing yeast to create a circular mycoplasma genome of nearly 600 kb in one step [22].

Traditional reverse genetics methods for RNA viruses, including coronaviruses, have faced challenges in assembling and preserving full-length molecular clones. To address this, researchers have turned to yeast homologous recombination systems for reverse genetics research. In 2020, a team led by Jorerg Jores and Volker Thiel developed a yeast-based synthetic biology method that established a rapid and stable reverse genetics platform for RNA viruses [23]. Building on this work, Pei Yongshi and colleagues successfully synthesized the cDNA of SARS-CoV-2 using yeast homologous recombination technology in 2021 [24]. This breakthrough has provided crucial support for understanding SARS-CoV-2 pathogenesis and developing prevention and control strategies. The advancement of virus genome synthesis technology also holds promise for applications in biopharmaceutical design [25], gene therapy [26,27], and oligonucleotide drug development [28].

In the rapidly advancing field of synthetic biology, researchers are not only focused on innovating DNA synthesis and splicing technology but also on scaling up and automating processes. The US Department of Energy Agile Biofoundry has created a web-based DNA assembly design software Version 2.2.2 (09-MAR-15) called j5, which can generate assembly diagrams and processes based on user input or recommended methods [29]. This software also provides instructions for automated pipetting workstations and microfluidics, significantly increasing design efficiency. The University of Edinburgh’s EGF facility in the UK is capable of completing over 2000 DNA assembly reactions per week, with a throughput 20 times faster than manual operations by researchers [30]. Although automated synthetic biology technology is still in its early stages, collaboration between researchers and engineers from various fields such as synthetic biology, automation, and analytical chemistry is essential to optimize facility platforms through multiple rounds of engineering iterations. This collaboration is crucial for supporting the rapid advancement of both basic and applied research in synthetic biology, potentially leading to groundbreaking innovations.

**Figure 1:**
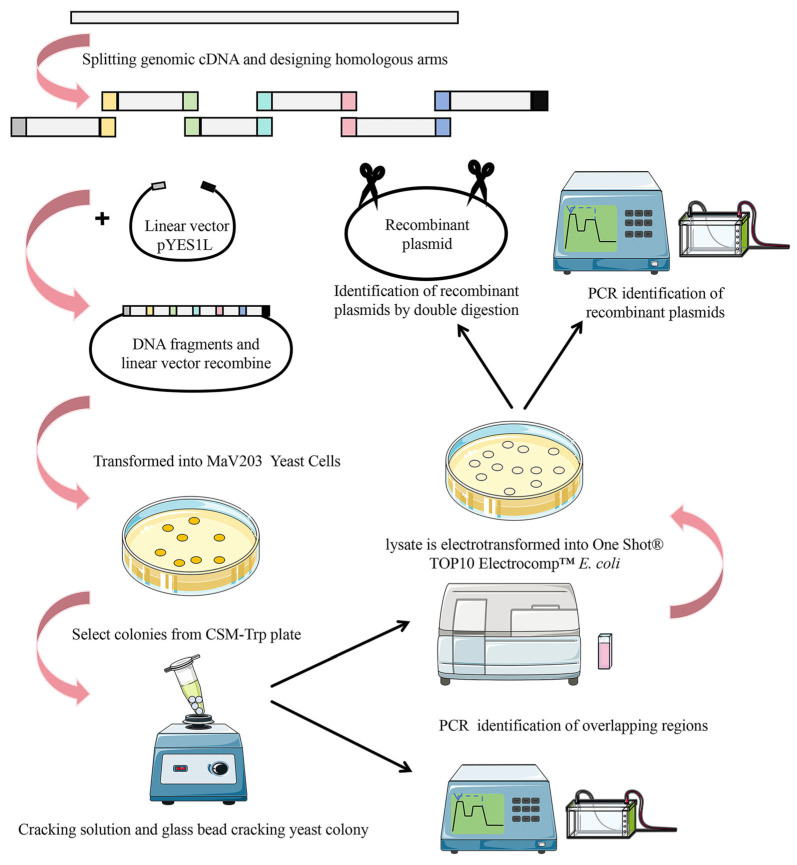
The experimental operation process of this study.

This study aimed to establish standardized program parameters for splicing DNA fragments of approximately 5 kb. The efficiency of homologous recombination is primarily influenced by the length of the homologous arm and the vector-to-fragment ratio. To achieve this, we utilized a plasmid containing the genome cDNA of severe acute respiratory syndrome coronavirus type 2 (NC_045512.2) as a template to fragment the cDNA into 5 kb fragments. By testing different homologous arm lengths and vector-to-fragment ratios, we determined the optimal parameters. The process involves splitting the virus genome cDNA, splicing large DNA fragments, amplifying recombinant plasmids, and identification (Figure 1). The optimal ratio identified in the study will serve as a reference for splicing DNA fragments of around 5 kb, with potential applications in automated splicing programs for DNA fragments of similar size. This research provides valuable insights for future studies on preventing and controlling viral epidemics through reverse genetics.

## 2. Results

### 2.1. Selection of Homologous Arms for cDNA Fragments from Viral Genomes

In the process of selecting homologous arms for viral genome cDNA fragments, we employed the 30 kb viral genome cDNA plasmid pUC-SARS-CoV-2 as a template. Specific primers were created to segment it into six DNA fragments of approximately 5 kb each, with overlapping sequences of 40 bp, 60 bp, and 80 bp between adjacent fragments for homologous recombination. The Tm values of homologous arm sequences of different lengths vary from 73.3 °C to 75.5 °C (Table S1).

### 2.2. Examining the Influence of Different Lengths of Homologous Arms and Various Fragment Carriers on Splicing Efficiency

**Figure 2:**
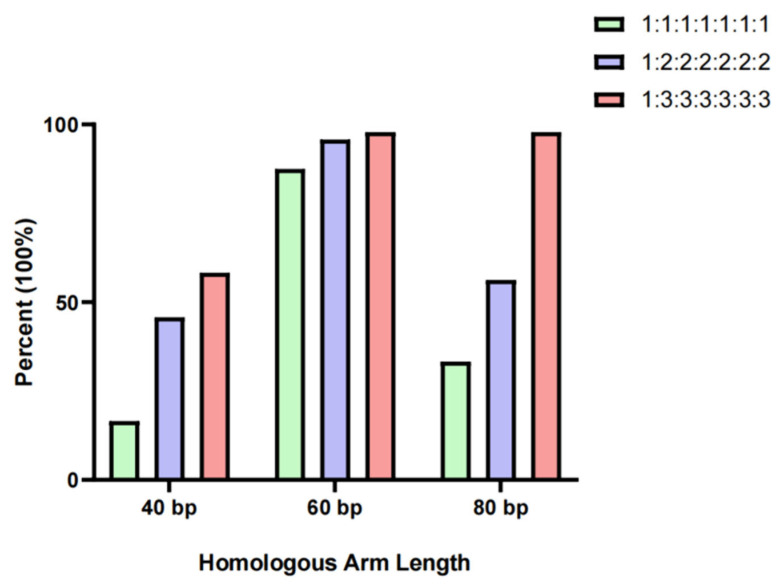
Recombination efficiency of different homologous arm lengths and vector fragment ratios.

We categorized the sizes of homologous sequences into three groups: 40 bp, 60 bp, and 80 bp for each recombinant fragment. We conducted homologous recombination experiments between the vector (100 ng) and the fragments using mass ratios of 1:1:1:1:1:1, 1:2:2:2:2, and 1:3:3:3:3. The results (refer to Table S2) showed that when the homologous sequence was 40 bp, the efficiency of homologous recombination increased as the ratio of the vector to each fragment increased, reaching 58.3% at its highest. For 60 bp homologous sequences, the recombination efficiency increased with the vector-to-fragment ratio, consistently exceeding 85% and peaking at 97.9%. However, with 80 bp homologous sequences, the ratio of vector to fragment had a significant impact on recombination efficiency. Increasing the ratio from 1:1:1:1:1:1 to 1:2:2:2:2:2 actually decreased efficiency and the number of clones, dropping below 48. However, at a ratio of 1:3:3:3:3:3, recombination efficiency reached 97.9% (Figure 2, Table S2).

### 2.3. Identification of the YAC Plasmid

**Figure 3:**
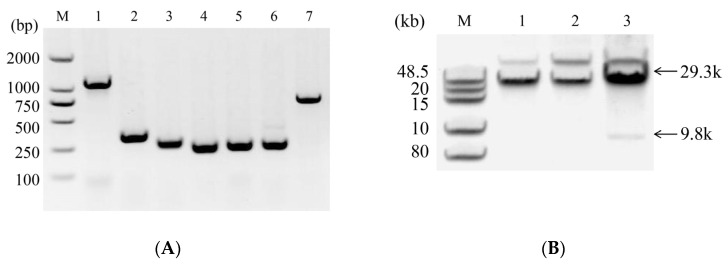
Identification of YAC plasmid. (**A**) PCR identification of YAC plasmid; M: 2K marker (BM101, TransGen Biotech, Beijing, China); 1–7: identification results of overlapping areas 1–7. (**B**) Identification results of double enzyme digested YAC plasmids; M: DNA marker (NEB, N3239S); 1: YAC plasmid with *Bam*HI-HF (NEB, R3136M); 2: YAC plasmid with *Sbf*I-HF (NEB, R3642S); 3: double enzyme digestion of YAC plasmid by BamHI-HF and SbfI-HF.

To identify the YAC plasmid, PCR and double restriction enzyme digestion methods are commonly used. However, the double restriction enzyme digestion method often requires a long time for pulse field electrophoresis separation. In this study, we aimed to optimize the electrophoresis conditions to reduce the time needed for identification. We used agarose gel electrophoresis, which is a faster method than pulse field electrophoresis. Monoclonal samples of *Escherichia coli* that were correctly identified by PCR were selected as templates for YAC plasmid digestion identification. The results of both PCR and digestion identification were found to be consistent (Figure 3).

## 3. Discussion

Since the emergence of SARS-CoV-2, there has been a growing awareness of the significant impact the virus has had on human society. Extensive research into the nature and transmission of the virus is essential for effectively managing outbreaks and safeguarding public health [31]. Reverse genetics is a crucial technology for studying viruses, and advancements in synthetic biology have led to the ability to synthesize viral genomes with increasing precision [32]. This allows for a better understanding of viral replication mechanisms, gene functions, and interactions with host cells, ultimately providing valuable insights for the prevention, diagnosis, and treatment of viral diseases. The synthesis of viruses began with the assembly of poliovirus and phiX174 bacteriophage using oligonucleotides [33,34], showcasing human capabilities in manipulating the microscopic world and paving the way for the synthesis of more complex organisms in the future. In 2008 and 2010, the Venter team achieved a significant milestone by successfully synthesizing the genomes of two types of mycoplasma [35,36], demonstrating continuous progress in virus synthesis technology.

Synthetic biology is a multidisciplinary field that involves designing, modifying, and synthesizing life. The complexity of living organisms often requires numerous trial-and-error experiments, leading to high research costs and slow progress. Automated synthetic biotechnology, with its automation, standardization, high-throughput capabilities, and advancements in information technology and artificial intelligence, is poised to revolutionize traditional biological research methods that rely on manual experimentation. This technology is expected to provide crucial technical and platform support for the rapid design and construction of microbial cell factories. Automated synthetic biotechnology has already made significant advancements in the rapid construction of microbial cell factories, particularly in the field of model strains, yielding impressive research outcomes [37,38,39,40].

Current research is actively striving to enhance the efficiency of yeast homologous recombination to minimize or eliminate errors. By optimizing the length of homologous arms and the vector-to-fragment ratio in yeast homologous recombination, researchers aim to improve the accuracy and effectiveness of DNA fragment splicing. This study is dedicated to enhancing the recombination efficiency of yeast homologous recombination technology, marking a crucial advancement in the realm of viral genome fragment splicing with substantial research implications.

In traditional DNA synthesis splicing, smaller fragments are typically synthesized using PCR between primers, slightly larger fragments are assembled using Gibson assembly, and larger fragments usually rely on the recombination system within the organism. In this study, we divided the viral genome cDNA fragment into approximately 5 kb fragments and utilized the 5 kb DNA fragment as the splicing unit to investigate the optimization of homologous recombination efficiency of large fragments in yeast. When selecting the length of homologous arms, we took into consideration the findings of previous researchers: Gibson suggested in 2011 that adjacent fragments in homologous recombination should have overlapping sequences of at least 40 bp [41], while Venter used a homologous arm length of 80 bp when synthesizing the genomes of two mycoplasma species in 2008 and 2010 [35,36]. Building on these insights, we set the parameters for homologous arm length at 40 bp, 60 bp, and 80 bp, respectively. It is important to note that despite the varying lengths of homologous arms, the Tm value remains consistent within a stable range (73.3 °C~75.5 °C). The Tm value was calculated using SantaLucia’s method and an online tool (https://www.biorun.com/tools/102.html, accessed on 1 September 2024) [42]. In essence, the efficiency of homologous recombination is primarily influenced by the length of homologous arms and the vector-to-fragment ratio.

In this study, we conducted extensive manual experiments and found that in most cases, the efficiency of recombination increases as the vector-to-fragment ratio decreases. Our results showed that yeast homologous recombination was most efficient when the vector-to-fragment ratio was 1:3:3:3:3:3. Additionally, we found that a 60 bp homologous arm length had the best effect, maintaining high assembly efficiency regardless of changes in the vector-to-fragment ratio, with homologous recombination efficiency remaining above 85%. The highest efficiency of homologous recombination, at 97.9%, was achieved with a 40 bp homologous arm length and a vector-to-fragment ratio of 1:3:3:3:3:3:3. However, there are two important points to consider. First, when the length of the homologous arm is 60 bp, there is a slight increase in recombination efficiency when the mass ratio of the vector to each fragment changes from 1:2:2:2:2:2 to 1:3:3:3:3:3. Out of 48 monoclonal antibodies tested, only one showed an increase in positivity, but the required fragment mass also increased by 1.5 times. This means that a higher concentration of fragments is needed, which can lead to increased costs in industrial-grade automated splicing production processes. Secondly, when the length of the homologous arm is 80 bp and the mass ratio of the vector to each fragment is 1:3:3:3:3:3:3, the homologous recombination efficiency remains high, at 97.9%. However, the longer homologous arm will result in increased costs for primer synthesis. Taking all factors into consideration, it is recommended to use a 60 bp homologous sequence with a vector-to-fragment ratio of 1:2:2:2:2:2 when using yeast homologous recombination to splice large segments of the virus genome.

## 4. Materials and Methods

### 4.1. Plasmids and Bacterial Strains

The following plasmids were used in this study: SARS-CoV-2 (NC_045512.2) viral genome cDNA plasmid: pYES1L-SARS-CoV-2 (constructed and preserved in the laboratory), pYES1L vector fragment (A13286 kit), purchased from Thermo company. The following strains were used: MaV203 yeast competent cells (A13286 kit); One Shot TOP10 Electrocomp *E. coli* (A13286 assay kit) was purchased from Thermo Fisher Scientific, Waltham, MA, USA, a company responsible for the induction of *E. coli* cells.

### 4.2. Sequence Splitting, Homologous Arm Design, and Primer Design Synthesis

The viral genome cDNA was split into fragments of around 5 kb, and then an online platform (https://www.biorun.com/tools/102.html, accessed on 5 September 2024) was created to determine the Tm values of the homologous arms. Utilizing pYES1L-SARS-CoV-2 as a template and SnapGene 4.3.6 software, primers were created to amplify genomic fragments according to the length of homologous sequences. Unique primers were formulated for distinct overlap regions to detect yeast homologous recombination products. These sequences were manufactured by Beijing Qingke Biotechnology (Beijing, China). Nuclease-free water was mixed with each primer powder to reach a concentration of 10 µM for each primer.

### 4.3. Amplification of Viral Genome Fragments and Homologous Recombination Splicing in Yeast

#### 4.3.1. Viral Genome Fragment Amplification

PCR amplification was carried out using pUC-SARS-CoV-2 (10 ng) as the template, with various specific primers to produce genome fragments of different lengths. The Q5 High Fidelity 2 × Master Mix (NEB, M0492L, Ipswich, MA, USA) was used for the PCR amplification process, following the instructions provided on the official website (https://www.neb.cn/zh-cn/protocols/2012/12/07/protocol-for-q5-high-fidelity-2x-master-mix-m0492, accessed on 5 September 2024).

#### 4.3.2. Yeast Homologous Recombination Splicing of Viral Genome Fragments

A DNA mixture was formed by combining the pYES1L vector and each recombinant fragment in different ratios: 1:1:1:1:1:1 (both vector and fragment are 100 ng), 1:2:2:2:2:2:2 (carrier 100 ng, each fragment is 200 ng), or 1:3:3:3:3:3:3 (vector 100 ng, each fragment is 300 ng). Once mixed, 100 μL of rapidly thawed MaV203 yeast competent cells was introduced separately for transformation.

Detailed transformation steps can be found in the Thermo A13286 reagent kit operation manual (https://assets.thermofisher.cn/TFS-Assets/LSG/manuals/geneart_highorder_genetic_assembly_man.pdf, accessed on 5 September 2024).

### 4.4. PCR Identification of Yeast Homologous Recombination Products

#### 4.4.1. Preparation of Yeast Cell Lysate Diluent

To begin, 10 µL of lysis buffer (provided in the A13286 kit) was added evenly into a sterile 96-well deep hole plate. Next, yeast monoclonal colonies from CSM-Trp agar plates were retrieved and placed in deep wells. Repeated blowing and suction were performed for approximately 1 min. Then, 5 µL of cell lysate was transferred to a sterile PCR tube and heated at 95 °C for 5 min in a PCR machine. It was allowed to cool at 4 °C for another 5 min. Next, 45 µL of nuclease-free water was added to each yeast cell lysate and mixed by pipetting 3–5 times. Finally, the remaining 5 µL of cell suspension was reserved at 4 °C for the subsequent transformation of yeast homologous recombination plasmid into competent cells of One Shot TOP10 Electrocomp *E. coli*.

#### 4.4.2. PCR System

Specific identification primers were designed on the basis of the overlapping region sequences, with a PCR system of 50 μL for each reaction, containing the following components: 1.0 µL of upstream primer (10 μM) and 1.0 µL of downstream primer (10 μM), along with 25.0 µL of Hot Start Taq 2× Master Mix (NEB, M0496S, Ipswich, USA), 22.5 µL of nuclease-free water, and 0.5 µL of yeast cell lysis dilution.

### 4.5. Evaluation of Genome Splicing Efficiency

A total of 48 monoclonal clones were selected from each group and identified following the procedure described in Section 4.4. The clones underwent electrophoresis separation on a 1% agarose gel, followed by the analysis of the gel images. A clone was deemed positive if the target band was visible, and the positivity rate was subsequently calculated. The accuracy, or positivity rate was obtained by dividing the number of positive clones by the total number of clones and multiplying by 100%.

### 4.6. Transformation and Identification of the YAC Plasmid into Escherichia coli

#### 4.6.1. Transformation of YAC Plasmid into *Escherichia coli*

The monoclonal antibody that tested positive was chosen, followed by lysing the 5 µL cell suspension from the reserved monoclonal antibody in Section 4.3.1. Finally, it was transformed into One Shot TOP10 Electrocomp *E. coli* competent cells following the transformation steps outlined in the Thermo A13286 kit operation manual (https://assets.thermofisher.cn/TFS-Assets/LSG/manuals/geneart_highorder_genetic_assembly_man.pdf, accessed on 5 September 2024). With electric shock parameters set at 2400 V, 200 Ω, and 25 μF, Gene Pulser Xcell (Bio Rad) delivers precise results.

#### 4.6.2. YAC Plasmid PCR Identification

To isolate YAC plasmids, the instructions in Thermo’s plasmid extraction kit (K210006) manual were followed, which can be found at the following link (https://assets.thermofisher.cn/TFS-Assets/LSG/manuals/purelink_hipure_plasmid_dna_purification_man.pdf, accessed on 5 September 2024). After extraction, the YAC plasmid was used as a template for PCR identification with specific primers for homologous recombination products. The PCR system and procedure were the same as those described in Section 4.4.2.

#### 4.6.3. Identification of YAC Plasmid Double Enzyme Digestion

*Bam*H I-HF (NEB, R3136V, Ipswich, USA) was utilized for the extraction of the YAC plasmid. Quantities of 1 μL of *Sb*f I-HF (NEB, R3642S, Ipswich, USA) each, 5 μL of rCutSmart, and 2 μg of YAC plasmid were combined with 50 μL of nuclease-free water. The reaction was allowed to proceed at 37 °C for 1 h, followed by thermal inactivation at 80 °C for 20 min. Electrophoretic separation was conducted on a 0.5% 1 × TAE agarose gel at 92 V for 3 h. The results were evaluated through gel imaging.

## 5. Conclusions

In this study, we utilized SARS-CoV-2 cDNA as a template to generate DNA assembly fragments approximately 5 kb in size. Through optimizing the length of homologous arms and the vector-to-fragment ratio in yeast homologous recombination, we successfully and efficiently completed the fusion of large viral genome fragments, which could be stably amplified in *E. coli*. Our findings revealed that in the yeast homologous recombination system, the highest recombination efficiency was achieved when using a 1:3:3:3:3:3 ratio of the vector to each fragment, regardless of the length of the homologous arm (40 bp, 60 bp, or 80 bp). Considering production cost and scalability, we recommend utilizing a 60 bp homologous arm length and a 1:2:2:2:2:2 vector-to-fragment ratio when automating the fusion of 5 kb viral genome fragments using the yeast homologous recombination system, as this approach can achieve high efficiency at a relatively low cost.

## Data Availability

Data are contained within the article.

## Supplementary Materials

The following supporting information can be downloaded at: https://www.mdpi.com/article/10.3390/ijms252413742/s1.
