# Supplementary material for: Optimizing Yeast Homologous Recombination for Splicing Large Coronavirus Genome Fragments
Source: Int J Mol Sci. 2024 Dec 23;25(24):13742. doi: 10.3390/ijms252413742 (PMC11677428; doi:10.3390/ijms252413742)
Supplement: Supplementary file 1 [file ijms-25-13742-s001.zip › supplementary/Table S1.docx]

**Table S1. Homologous arm sequence and Tm value.**

| **Homologous arm length** | **Sequence** | **Tm value**  **(℃)** |
| --- | --- | --- |
| 40 bp | CCGCGCTGATACCGCCGCATTAAAGGTTTATACCTTCCCA | 75.4 |
|  | CCTCCACACGCAAGTTGTGGACATGTCAATGACATATGGA | 73.3 |
|  | CCAACCACCACAAACCTCTATCACCTCAGCTGTTTTGCAG | 74.3 |
|  | GCCGCCACTAGAGGAGCTACTGTAGTAATTGGAACAAGCA | 73.3 |
|  | GGGTGTGGACATTGCTGCTAATACTGTGATCTGGGACTAC | 73.3 |
|  | CCTCGTGAAGGTGTCTTTGTTTCAAATGGCACACACTGGT | 73.9 |
|  | CCTCGCCGCAGTTAATTAAAGTCAGTGAGCGAGGAAGCGC | 75.6 |
| 60 bp | CCGCATTAAAGGTTTATACCTTCCCAGGTAACAAACCAACCAACTTTCGATCTCTTGTAG | 74.9 |
|  | GAGAGAAGTGAGGACTATTAAGGTGTTTACAACAGTAGACAACATTAACCTCCACACGCA | 74.5 |
|  | AAGTTGAGGGTTGTATGGTACAAGTAACTTGTGGTACAACTACACTTAACGGTCTTTGGC | 74.7 |
|  | GGTGGCTGTATTAATGCTAACCAAGTCATCGTCAACAACCTAGACAAATCAGCTGGTTTT | 74.9 |
|  | CATCTGTAGGTCCCAAACAAGCTAGTCTTAATGGAGTCACATTAATTGGAGAAGCCGTAA | 74.8 |
|  | GACCGCCTCAATGAGGTTGCCAAGAATTTAAATGAATCTCTCATCGATCTCCAAGAACTT | 75.5 |
|  | CGCATCTGTGCGGTATTTCACACCGCATAGATCGGCAAGTGCACAAACAATACTTAAATA | 75.2 |
| 80 bp | ATTAAAGGTTTATACCTTCCCAGGTAACAAACCAACCAACTTTCGATCTCTTGTAGATCTGTTCTCTAAACGAACTTTAA | 74.4 |
|  | GGACAACAGTTTGGTCCAACTTATTTGGATGGAGCTGATGTTACTAAAATAAAACCTCATAATTCACATGAAGGTAAAAC | 74 |
|  | ATGACGTAGTTTACTGTCCAAGACATGTGATCTGCACCTCTGAAGACATGCTTAACCCTAATTATGAAGATTTACTCATT | 75.2 |
|  | AAAACGTAATGTCATCCCTACTATAACTCAAATGAATCTTAAGTATGCCATTAGTGCAAAGAATAGAGCTCGCACCGTAG | 74.3 |
|  | CTCACTGTCTTTTTTGATGGTAGAGTTGATGGTCAAGTAGACTTATTTAGAAATGCCCGTAATGGTGTTCTTATTACAGA | 74.4 |
|  | TACATCACCAGATGTTGATTTAGGTGACATCTCTGGCATTAATGCTTCAGTTGTAAACATTCAAAAAGAAATTGACCGCC | 75.4 |
|  | TATTTTCTCCTTACGCATCTGTGCGGTATTTCACACCGCATAGATCGGCAAGTGCACAAACAATACTTAAATAAATACTA | 74.6 |
