# Supplementary material for: Optimizing Yeast Homologous Recombination for Splicing Large Coronavirus Genome Fragments
Source: Int J Mol Sci. 2024 Dec 23;25(24):13742. doi: 10.3390/ijms252413742 (PMC11677428; doi:10.3390/ijms252413742)
Supplement: Supplementary file 1 [file ijms-25-13742-s001.zip › supplementary/Table S2.docx]

**Table S2. Statistical Table of Reorganization Efficiency of Each Group**

| Group | Homologous sequence level | Mass ratio of carrier to each fragment | Number of positive samples | Total number of samples | Positive rate |
| --- | --- | --- | --- | --- | --- |
| 40-1 | 40 bp | 1:1:1:1:1:1:1 | 8 | 48 | 16.6% |
| 40-2 |  | 1:2:2:2:2:2:2 | 22 | 48 | 45.8% |
| 40-3 |  | 1:3:3:3:3:3:3 | 28 | 48 | 58.3% |
| 60-1 | 60 bp | 1:1:1:1:1:1:1 | 42 | 48 | 87.5% |
| 60-2 |  | 1:2:2:2:2:2:2 | 46 | 48 | 95.8% |
| 60-3 |  | 1:3:3:3:3:3:3 | 47 | 48 | 97.9% |
| 80-1 | 80 bp | 1:1:1:1:1:1:1 | 16 | 48 | 33.3% |
| 80-2 |  | 1:2:2:2:2:2:2 | 27 | 48 | 56.25% |
| 80-3 |  | 1:3:3:3:3:3:3 | 47 | 48 | 97.9% |
